# Retrospective narratives of COVID-19 experiences: a qualitative study of written accounts by people in public in the context of a performative intervention

**DOI:** 10.1186/s12889-026-28976-9

**Published:** 2026-08-14

**Authors:** Till Neugebauer, Amand Führer, Maren Schuster, Anne Marx, Franziska Waldschmitt, Yüce Yilmaz-Aslan, Patrick Brzoska

**Affiliations:** 1 Health Services Research, School of Medicine, Faculty of Health, Witten/Herdecke University, Alfred-Herrhausen-Straße 50, Witten, 58455 Germany; 2 Institute for Medical Epidemiology, Biometrics and Informatics, Interdisciplinary Center for Health Sciences, Martin Luther University Halle-Wittenberg, Halle/Saale, Germany; 3 Section of Media and Communication Studies, Department of Media, Music, and Speech Studies, Martin Luther University Halle-Wittenberg, Halle/Saale, Germany; 4 Kollektiv Kubik e.V., Weimar, Germany

**Keywords:** COVID-19, Qualitative research, Public engagement, Performative research, Participation

## Abstract

**Background:**

This qualitative study explored how people retrospectively described their experiences of the COVID-19 pandemic. It aimed how these experiences are remembered and interpreted over time.

**Methods:**

A qualitative study design was employed, based on short texts written by members of the public during a performative intervention in public space. The texts were thematically analyzed to identify common experiences and emotions.

**Results:**

The findings show that participants described the pandemic not only as a health crisis but also as a major social and emotional challenge. Feelings of fear, uncertainty, and loss of control were common, alongside attempts to find meaning and emotional stability. Narratives also suggest that the ability to reflect on the pandemic as a period of adaptation or personal growth depended on access to stable resources such as housing, social support, and digital connectivity.

**Conclusions:**

Overall, the study highlights that how the pandemic is remembered is closely linked to social conditions, underlining the importance of inclusive and reflective approaches to understanding the long-term impacts of COVID-19. In addition, it demonstrates that social inequalities in both the interpretation of past crises and the development of inclusive strategies are important for future public health response.

**Supplementary Information:**

The online version contains supplementary material available at 10.1186/s12889-026-28976-9.

## Background

The COVID-19 pandemic had far-reaching effects on health across the globe and the lives of all population groups. In addition to morbidity and mortality, the pandemic reshaped everyday routines, social relations, emotional wellbeing, and perceptions of security, control, and belonging across societies [1]. Measures implemented to contain the spread of SARS-CoV-2, including contact restrictions, lockdowns, and limitations on mobility, deeply affected how people interacted with one another and how they experienced time, space, and social participation [2, 3].

While a substantial body of research has focused on infection dynamics, health system responses, risk communication, and social inequalities during the acute phases of the pandemic, considerably less attention has been paid to how individuals retrospectively reflect on and narrate their experiences once the immediate crisis has subsided [4, 5]. However, such perspectives are essential for assessing the longer-term impact of the pandemic in the context of lived experience and for understanding how meanings attributed to the crisis continue to evolve over time.

During the pandemic, public perception was shaped by misinformation in social media, mistrust of established media outlets, knowledge gaps and uncertainty, restrictions on fundamental rights, the role of authorities, and negative economic consequences [6]. Importantly, the pandemic was not experienced or remembered as a uniform event. Studies have shown substantial variation in how individuals perceived risks, controllability, and personal impact, depending on social position, cultural background, and biographical context [7, 8]. However, rather than isolating predefined social groups, examining open narrative expressions allows insight into how people themselves frame what was meaningful, distressing, or transformative about the pandemic.

Narrative self-expressions offer a particularly valuable lens for capturing the emotional and social dimensions of crisis experience [9]. They can convey affective states, moral evaluations, and implicit assumptions that may remain inaccessible through standardized instruments. In recent years, scholars have emphasized the importance of memory cultures and participatory forms of remembrance in the aftermath of collective crisis [10, 11]. Such approaches underline the relevance of public spaces and performative, low-threshold interventions as sites where individual memories can be articulated in relation to broader social discourse. Public interventions enable spontaneous, self-directed expressions that are shaped by everyday contexts rather than institutional research settings and thus offer unique insights into how collective crises are remembered, emotionally processed, and communicated beyond formalized narratives of crisis management or risk communication [5].

## Methods

### Aim and relevance of the study

The present study builds on this perspective and aimed to qualitatively analyze written narratives generated through a public participatory intervention to explore how people retrospectively describe their experiences, emotions, and perceptions related to the COVID-19 pandemic. Moreover, the analysis sought to identify the dominant categories emerging from the texts and to interpret them, from a social constructionist perspective, which understands meanings and accounts as socially, historically, and linguistically situated constructions, in relation to broader processes of emotional processing, collective memory, and social reflection [12].

This analysis contributes to understanding the pandemic not only as an epidemiological phenomenon but also as an emotional and social event. It highlights how individual voices can open new perspectives on health, belonging, and participation in society. The study thus provides a contribution to the critical reflection on health communication, participation, cultures of remembrance in the context of the COVID-19 pandemic, and resulting public health responses.

### Study design and setting

A qualitative research design was employed to analyze written secondary data from the performative public intervention *Message in a Bottle*. The intervention took place on March 22, 2025, in the pedestrian area of downtown Halle (Saale), Germany, and was subsequently presented as part of the exhibition “Stuck in Mobility – Seafarers and Refugees during the Pandemic”, which highlighted the lived experiences of social groups in vulnerable conditions during the COVID-19 pandemic [13]. The intervention symbolically drew on the notion of a message in a bottle as a communication from isolation and invited passers-by to reflect on their own experiences of the COVID-19 pandemic. By fostering personal reflection, it aimed to raise awareness of the challenges faced by the groups represented in the exhibition and to encourage engagement with the exhibition itself. Although embedded within the exhibition, the present analysis is based exclusively on the narratives collected during the public intervention. As participants reflected on their own experiences before any engagement with the exhibition, the resulting narratives constitute an independent qualitative dataset and were analyzed separately from the exhibition’s thematic focus. The exhibition itself and the experiences of seafarers and refugees presented therein were therefore not part of the dataset.

The specific location in public space was chosen as it represents a highly frequented and central area of the city center. The proximity to retail facilities, pedestrian routes connecting local and long-distance transport, as well as situations of lingering in the market square or waiting for the next tram were decisive factors in this choice. The public setting thus functioned as both a social and communicative context that shaped the character of the responses, which were spontaneous, emotionally charged, and closely embedded in everyday life.

### Data collection

Data were collected by two members of the artist collective *Kollektiv Kubik*, a group of performance artists specializing in public art interventions. The intervention was originally conceived as a performative element of the exhibition, and the resulting written narratives were subsequently analyzed as secondary qualitative data. Participation was open, voluntary, anonymous, and based on self-selection among passers-by. As the study focused on written narratives of pandemic experiences rather than differences between population groups, no personal or sociodemographic information was collected. Consequently, the unit of analysis was the written narrative rather than the individual participant.

Participants were invited to write short self-reports on provided writing paper, detailing their experiences, perceptions, and emotions related to the COVID-19 pandemic. The guiding question was deliberately open and minimal: *“How did you feel during the pandemic?”* and was asked both in English and German. The format was non-standardized, allowing contributors to freely determine topic, language, and length. This openness reflects the performative and participatory nature of the intervention.

Data were collected during the opening event intervention and over the subsequent 4 weeks. In total, 101 texts were gathered, digitized, and transcribed for analysis. For the present study, these texts were subjected to a secondary qualitative analysis. After composing their message in a bottle, participants were invited to visit the exhibition, where they could also open messages written by unknown others. Admission to the museum hosting the exhibition was free of charge during the opening event. Field notes were kept throughout the intervention to document contextual aspects of the data collection process, including participation dynamics, group composition, site-specific characteristics, the sequence of events, self-reported patient characteristics and observed participant reactions. These notes served to contextualize the narratives in their original performative and public setting [see Appendix A].

### Data analysis

Data analysis followed the principles of qualitative content analysis according to Kuckartz and Rädiker [14]. This method is a rule-based approach that combines deductive category development derived from theoretical considerations with inductive refinement based on the empirical material. The analysis proceeds in iterative coding cycles, allowing categories to be continuously reviewed, differentiated, and adapted while ensuring transparency and methodological rigor. Given that the material originated from a performative intervention and consisted of short, heterogenous narratives, an open coding approach to category development was applied. The process was informed by a social constructionist perspective, according to which written narratives are not treated as transparent or context-free representations of past experiences. Rather, they are understood as socially, historically, and situationally embedded accounts through which individuals construct and negotiate meaning [12, 15]. For the interpretation of the texts, this meant that the categories were understood as expressions of retrospective meaning-making, showing how contributors constructed the pandemic as an emotional, social, and political experience in relation to their perceived resources, relationships, and social conditions. This perspective guided the interpretation of the categories that emerged from the qualitative content analysis, while the analytical procedure itself followed the rule-based approach proposed by Kuckartz and Rädiker [14].

In a first step, all texts were read and coded by the first author. Afterwards a second coding was performed by artificial intelligence (AI) through inductive category formation. Recent methodological literature suggests that large language models (LLM) can support qualitative analysis by enhancing coding efficiency, consistency and transparency while facilitating the identification of patterns across complex textual datasets [16, 17]. At the same time, it is recommended that LLMs should be regarded as complementary analytical tools rather than substitutes for qualitative researchers, as contextual understanding, reflexivity, theoretical interpretation, and methodological responsibility remain with the research team [18]. This hybrid approach can therefore combine the computational strengths of LLMs with the interpretative expertise required for rigorous qualitative research [19].

In the present study, a LLM was used in terms of code validation, complementing the initial manual coding. The LLM was used to support consistent pattern detection across the dataset and to complement the initial manual coding through predefined analytical parameters, without replacing human interpretive judgment. For the AI-assisted coding process a local offline version of *OpenAI’s gpt-oss 20B* was used. Access to and interaction with the LLM was enabled via LM Studio 0.3.30. For the inductive category formation, the LLM was configured with a temperature setting of 0.1 to ensure reproducibility while maintaining a minimal level of creative variation. The temperature value, ranging from 0 to 2, determines how closely the LLM’s output adheres to the input text [20]. Lower values lead to more deterministic and consistent results, while higher values increase variability but risk incoherence. The analysis prompt included the instruction for inductive category development and the study’s research aim: „Conduct an inductive coding of the following document [Message in a Bottle Transcripts]. The goal is to understand how people retrospectively narrate their experiences, emotions, and perceptions regarding the COVID-19 pandemic.“ The initial AI-assisted coding process required 29.91 seconds.

The two coding systems — AI-assisted and manual — were compared and tested for intercoder reliability using Cohen’s Kappa [21]. Agreement reached κ = 0.61, indicating high reliability. In cases of divergence, categories were refined and redefined. The entire dataset was then recoded by the LLM using the revised coding scheme.

Subsequent analysis and interpretation of the results were carried out collaboratively by the research team and are presented below.

### Ethical considerations

Participation was voluntary and anonymous, and no identifying information was collected. Participants were informed about the publication of their responses as part of the exhibition. Support was provided to individuals with limited literacy or non-native language backgrounds while ensuring self-determined participation.

The AI-assisted analysis was conducted using a locally operated, offline LLM, which prevented any transfer to external servers or third parties. This approach minimized risks related to privacy, data security, and unintended data storage. The research team maintained full interpretive responsibility and critically reviewed the model’s outputs, ensuring transparency and methodological integrity. As this study uses secondary data, no ethics approval was needed for the analysis process.

## Results

The analysis comprised 101 written narratives, each representing the account of one participant. During the opening event, approximately 45 interactions with members of the public were documented, while additional narratives were collected over the following four weeks. As participation was anonymous and no personal or sociodemographic information was collected, the participant group is described on the basis of field observations. These indicated a heterogeneous group of passers-by, including individuals with varying educational backgrounds, literacy levels, and German language proficiency. At the same time, the majority appeared to be young adults (approximately 25–35 years) who were culturally and socially engaged and perceived the topic as personally relevant. Alongside individuals, small groups and couples participated spontaneously after being approached in the public space or having deliberately sought out the stand. The field notes further show that some participants stated that they rarely visit museums but were drawn in by the open and accessible nature of the intervention. This included people who described themselves as less academically inclined or with lower formal education. Five participants had difficulties with reading and writing, and their contributions were made with assistance.

The participants’ perceptions and experiences, as shared by them in their messages, can be grouped into four central dimensions: (1) Psychological and emotional dimension (2) Social dimension (3) Education, work, and social participation (4) Interplay of health and political perceptions.

### Psychological and emotional dimension

Participants frequently described fear and anxiety as major elements of daily life during the pandemic. Beyond the fear of personal infection, many expressed deep concern about potentially transmitting the virus to family or friends.

“For me, the beginning of the pandemic was a constant alternation between the feeling of deceleration and calm, but also the enormous fear for my family. This fear — perhaps also of being responsible for the infection or severe illness of a loved one — has never left me over the years.” (FP-5)

This sense of helplessness was referred to by one participant as *“powerlessness”* (FP-9), often evolving into a lasting anxiety about navigating social life. Some also feared the collapse of public life.

The effects on emotional wellbeing were also linked to government containment measures. Younger participants, in particular, expressed frustration and anger about what they perceived as the loss of their youth. This loss reflected a broader uncertainty about “the lost years” (FP-8).

At the same time, several participants reported that the pandemic offered them an opportunity for self-reflection and introspection. Amidst stillness and isolation, they found space to reconsider their values and life goals:

“Since I was alone a lot (my parents could still go to work), I had a lot of time to think about myself and got to know myself anew. That really helped me.” (FP-45)

Others described a sense of emotional stabilization, achieved by staying in safe environments or creating new routines. Overall, the emotional tone was ambivalent — between relief through deceleration and strain through loneliness, between new inner calm and persistent uncertainty about the future.

### Social dimension

A central topic across the messages was the experience of closeness and distance in social relationships. Many participants described the period as marked by loneliness, isolation, and a loss of social connectedness:

“The time without social contact — at work and in my free time — was extremely stressful and quite suffocating. I felt alone in many moments.” (FP-13)

Others used words like *“locked in”* (FP-1) or *“excluded”* (FP-9), reflecting the breakdown of familiar forms of community and everyday interaction. For many, the lack of physical closeness — especially due to hospital and nursing home visitor restrictions — was particularly painful.

At the same time, new forms of cohesion emerged within smaller social circles. Family and friendship were often experienced as stabilizing factors that helped participants cope emotionally. However, the messages also revealed how the pandemic deepened existing inequalities and conflicts. Diverging political opinions and risk perceptions within families and friend groups became additional sources of tension that, for some, persist to this day:

“At the same time, part of my family started doubting the measures and especially the politics behind them. My grandparents, who helped raise me, thought the restrictions were exaggerated. Sadly, this caused an emotional distance between us.” (FP-24)

These tensions highlight the ambivalent social dynamics of the pandemic, where some relationships deepened, others were permanently fractured.

### Education, work, and social participation

Education and work environments were key fields of experience during the pandemic. Students and pupils described major challenges associated with online learning, performance pressure, and a lack of social integration: “My studies were online — I think I understood very little.” (FP-2).

Such conditions led to feelings of stagnation or regression. Many spoke of “missed opportunities,” “lost experiences,” and “stolen youth” (e.g., FP-8, FP-46).

However, others viewed the situation as a chance for self-directed learning and flexibility: “I actually loved homeschooling because I could organize my day however I wanted.” (FP-7).

In the workplace, differences in living situations became especially apparent. Some participants continued working from home and even benefited from a slower pace of life, while others faced unemployment, financial anxiety, or severe psychological strain in so-called “essential jobs.”

Many also described a withdrawal from public spaces, limiting opportunities for cultural and civic participation — such as traveling, celebrating, or engaging in political activities. This interruption of social routines was often experienced as a deep rupture in everyday life and identity.

### Interplay of health and political perceptions

Health was a recurring topic encompassing both physical and psychosocial dimensions. Participants recounted personal infections or illnesses among relatives, often associated with isolation and fear for loved ones:

“I felt terrible because my mother passed away and I couldn’t say goodbye or attend her funeral due to the long distance to Munich!” (FP-3)

Alongside such personal losses, participants also reported health strains from lack of movement, loneliness, or perceived side effects of vaccination. Health was framed not only as a physical condition but as a central marker of control over one’s life.

In many messages, health perceptions intertwined with political evaluations. Trust in institutions and subjective assessments of risk were deeply interconnected. While some viewed public health measures as necessary and reasonable, others criticized them as “disproportionate,” “excessive,” or “authoritarian” (e.g., FP-22, FP-58, FP-70). Where measures were perceived as arbitrary or inconsistent, mistrust and skepticism grew:

“I thought, how can all of humanity be fooled like that? There were so many deaths from ‘medicines’ and vaccines. I said right away — that thing came from a lab.” (FP-71)

These narratives mirror the broader social polarization that continues to resonate today.

Yet, many participants also re-evaluated health as a renewed core value: “I appreciate health and freedom more than ever — and we all should!” (FP-13).

Across numerous statements, there was a shared wish for politicians to learn how they can improve their crisis management in the future. Accordingly, a reflective approach to pandemic-related political processes may strengthen trust in governmental measures and enable a more rapid response to future challenges by drawing on the experiences already gained. The pandemic thus appears as a double experience — both a health challenge and a test of trust, solidarity, and collective responsibility.

## Discussion

This qualitative study examined how people retrospectively narrate their experiences of the COVID-19 pandemic. From a social constructionist perspective, these narratives can be understood as socially situated accounts through which participants retrospectively construct meaning around their pandemic experiences [12]. They reflect a complex interplay of individual emotions, social dynamics, structural conditions, and collective interpretations of the pandemic. While dimensions such as emotional strain, social disruption, and adaption correspond to international findings from studies conducted during the pandemic [22–25], this study highlights how retrospective accounts shift the focus from the acute crisis to processes of memory and meaning-making. Their lived reality during the pandemic was characterized by an oscillation between emotional strain, social detachment, and efforts to find personal meaning and structure. Fear, loss of control, and concern for the future emerged as central components of memory. Similar patterns were reported in early-pandemic studies, in which many people expressed less fear for their own health and more concern about social cohesion and collective wellbeing [26, 27]. Importantly, these narratives also reflect processes of selective remembrance, highlighting which aspects of the pandemic are retained, re-evaluated, or given meaning over time. Positive reinterpretations appeared to be socially constructed, as they were closely linked to the availability of material and psychological resources [12]. The narratives suggest that individuals embedded in more protected and socially supportive environments may have been less exposed to some of the disruptive consequences of the pandemic, or may have had greater opportunities to reinterpret them as manageable and meaningful. These findings support the assumption that social position and group belonging can shape both the perception of a crisis and the ways in which it is remembered. This was already evident during the pandemic, as risk perception and psychological distress were unequally distributed across social groups. People with self-stated higher education levels and stable social resources more often reported coping, trust, and self-efficacy, whereas those with fewer material, social, or psychosocial resources more often experienced uncertainty and loss of control [28, 29].

In line with these findings, participants in the present study described phases of self-reflection, deceleration, and reorientation. This ambivalent experiential structure suggests that the pandemic is not solely remembered as a crisis but also as a period of adaptation and self-stabilization. However, deceleration did not carry a fixed meaning across the accounts. While some contributors retrospectively constructed it as an opportunity for self-reflection, emotional stabilization, or personal growth, others described the disruption of everyday life in terms of isolation, stagnation, exclusion, or loss of control. These different meanings appeared to be closely linked to conditions of relative security, such as access to housing, digital connectivity, social stability, and the ability to treat time as a resource for personal growth. Under such circumstances, experiences of loss of control could be reinterpreted as self-determination and meaning-making, supporting the notion of socially stratified differences observed in previous research [7, 8, 28]. From this perspective, socially constructed pandemic memories are not neutral accounts of a defined event, but reflect unequal opportunities for coping, sense-making, and narrative reappraisal. It can be argued that not only remembrance and interpretation of pandemic experiences, but also the meanings attributed to them, are socially constructed and shaped by broader social conditions (e.g. material and psychosocial resources [1, 2]). The retrospective framing of the pandemic as both crisis and opportunity may thus itself reflect a privileged position. While the suffering and disruption associated with the pandemic remained visible, many public contributors simultaneously reframed this period as one of autonomy, self-reflection, and personal growth. Inclusive processes of remembrance are therefore needed to help ensure that the experiences of socially disadvantaged groups and people living in vulnerable conditions are not marginalized in post-pandemic reflection and future public health preparedness [11]. This is particularly relevant because living conditions, access to information, and social support structures shape the extent to which individuals are able to develop coping mechanisms such as withdrawal, self-organization, and digital networking, and later talk about them [30]. If these conditions are not taken into account, post-pandemic reflection risks reproducing the same inequalities that structured pandemic experiences themselves. As a result, needs and living conditions of groups in vulnerable living conditions may remain insufficiently addressed in preparation for future crises.

### Implications for public health policy and pandemic preparedness

The findings suggest that public health response cannot be assessed solely through epidemiological indicators or health system performance. Environmental and social perceptions by the public fundamentally shape how pandemic measures are experienced, remembered, and evaluated [31]. It appeared that emotional responses such as fear, uncertainty, and powerlessness are not peripheral effects but integral dimensions of crisis governance. Preparedness strategies should therefore systematically integrate mental health support, transparent communication about uncertainty, and accessible psychological resources [32]. At the same time, coping capacities were closely tied to social and material conditions. The ability to frame the pandemic as a period of self-reflection or adaption depended on stable housing, secure income, digital access, and supportive relationships. Pandemic preparedness must therefore extend beyond biomedical infrastructure and address structural inequalities that condition resilience. Social protection, educational continuity, and equitable digital access are core components of effective public health response [33, 34].

The narratives also reveal lasting tensions around political decision-making. Perceptions of proportionality and transparency influenced levels of trust and social cohesion. Participatory formats that enable public dialogue during and after crises may strengthen legitimacy and reduce polarization [35]. A good example is the performative intervention used in this study. Mechanisms for inclusive evaluation and community engagement should be embedded in preparedness planning.

Finally, collective memory plays a critical role in policy learning [11]. If post-pandemic reflection privileges socially advantaged perspectives, existing inequalities could risk being reproduced in future responses. Inclusive processes of remembrance and evaluation therefore seem to be essential to ensure that marginalized experiences inform preparedness strategies. Taken together, the findings support a relational understanding of public health response [36]. Trust, solidarity, and social cohesion function as public health resources that require deliberate cultivation. Sustainable and equitable preparedness must integrate social, emotional, and political dimensions alongside epidemiological and logistical capacities.

### Strengths and limitations

The study has several strengths. The performative, arts-based approach facilitated spontaneous and self-directed narrative expressions within a public setting, allowing participants to contribute outside formal research environments. This approach may have encouraged emotionally rich and personal meaningful accounts. Conducting the intervention in public space further facilitated low-threshold participation and may have broadened participation by engaging individuals who are less likely to participate in qualitative research. The combination of manual and AI-supported coding allowed for a systematic and transparent analysis of heterogeneous and unstructured text material. In this context, AI-assisted coding served as a complementary tool to enhance consistency in pattern detection across the dataset, while human interpretation ensured contextual sensitivity and analytical depth. This hybrid approach enabled both breadth and interpretive rigor in the analysis [19, 37].

However, the findings must be interpreted within the exploratory nature of the study. Participation was voluntary and anonymous, likely resulting in selective engagement. Based on existing research, participation in such formats may be more common among individuals with higher levels of education, reflexivity, and social stability, potentially biasing the perspectives represented [38]. The open format precluded the systematic collection of sociodemographic data, limiting subgroup analyses.

In addition, although AI-assisted coding was used only as a secondary step to support validation and pattern identification after the initial researcher-led coding, it may not fully capture subtle meanings, contextual nuances, or culturally embedded expressions. To address this, all AI-generated outputs were critically reviewed and interpreted by the research team through a social constructionist perspective, focusing on how participants retrospectively constructed meaning around their pandemic experiences. Finally, consistent with this perspective, the findings should be understood as interpretations of socially situated narratives rather than objective representations of lived experience. The retrospective and situational nature of the narratives further limits transferability to other populations and contexts.

## Conclusion

This study offers a nuanced retrospective view of how individuals remember and interpret their experiences during the COVID-19 pandemic. Through a performative and publicly accessible format, the project made personal voices visible that are often absent from health research. The findings illustrate the ambivalence of pandemic memories, shaped by emotional strain, disrupted social relations, and efforts to regain meaning and stability. They also highlight how experiences of control, security, and coping were tied to social resources, underscoring the importance of considering structural inequalities when examining pandemic narratives.

The study demonstrates the value of integrating participatory public engagement with qualitative health research. Such approaches can enrich collective reflection, support inclusive health communication, and contribute to a more differentiated understanding of pandemic experiences in the post-pandemic period.

## Supplementary Information

Supplementary Material 1.

## Data Availability

Data from the Message in a Bottle intervention are available as part of the exhibition “Stuck in Mobility – Seafarers and Refugees during the Pandemic”.

## Abbreviations

AI: Artificial intelligence
LLM: Large language model
