# Supplementary material for: Retrospective narratives of COVID-19 experiences: a qualitative study of written accounts by people in public in the context of a performative intervention
Source: BMC Public Health. 2026 Aug 14;26:2364. doi: 10.1186/s12889-026-28976-9 (PMC13474965; doi:10.1186/s12889-026-28976-9)
Supplement: Supplementary file 1 [file 12889_2026_28976_MOESM1_ESM.docx]

**Retrospective narratives of COVID-19 experiences: A qualitative study of written accounts by people in public in the context of a performative intervention.**

**Appendix A. Field notes and quantitative overview during the opening event.**

**Participation**

- approx. 45 conversation partners/contacts
- 2–3 short periods during which no one stopped, each lasting no longer than 10 minutes
- In most cases, a personal initial contact was established before participants wrote down their response. They took considerable time to formulate their texts, and the responses were detailed.

**Diversity of participant groups**

- Individuals who were out shopping
- Occasional older men
- Young people interested in culture and socio-political issues (majority) (ages 25–35)
- Small groups and couples
- Male participants aged 30–40
- People who rarely visit museums but felt addressed by the action and its topic, with limited formal educational background (note: the original term was perceived as problematic by the author)
- 5 individuals who experienced difficulties with reading and writing (responses were written down for them)
- People with limited proficiency in the German language
- Former mayor of the city in which the intervention took place (did not want to be identifiable by his handwriting)
- Several individuals living in Halle-Neustadt (Considered a socially disadvantaged and deprived residential area outside the city center, where there is a high level of poverty and unemployment)
- A couple in which only one person is able to read and write, who had never visited the city museum before; they noticed the project during a city walk and subsequently visited the exhibition (question raised as to whether the use of simple language in the exhibition facilitates accessibility)
- Two men who arrived individually and stopped very purposefully at the poster or moved directly toward it
  - one of them was very enthusiastic, as the topic and his experiences during the pandemic seemed to occupy him deeply and he strongly identified with the question
  - the other began writing immediately
- People who came directly to the stand after attending the exhibition opening
- One person who had personal experiences of displacement during the pandemic and was pleased to share their story, reporting that they often do not feel welcome in Germany
- Occasional children who asked what was happening during the setup and the activity; their parents subsequently approached the stand
- Some children were too young to remember the pandemic period; however, they were mostly accompanied by older siblings who then participated.
- A woman visiting Halle who was specifically looking for exhibitions and was very pleased to receive the recommendation.

**Choice of location**

- Even during setup, the stand attracted curious looks and individual passers-by approached us
- Only a few minutes after setup, the first visitors arrived, including a small group that went directly into the exhibition afterward
- The stand was located directly along a main pedestrian flow, resulting in a continuous stream of passers-by around us

**Process and reactions**

- Many people initially stopped at the poster and read the text
- Almost all of them subsequently filled out a message in a bottle
- Some approached us directly and initiated conversation
- 10–15 people went into the exhibition immediately after visiting the stand
- Approximately ten additional people stated their intention to visit at a later time
- Overall very positive feedback from passers-by
- No encounters with discriminatory statements
- Some individuals chose not to write a message in a bottle, citing reasons such as having “nothing interesting to share” or that “they had been doing fine”; some could be convinced that positive experiences are also valuable and worth sharing

**Research question**

- The topic was relatable for all people
- Possibly formulated too openly for individuals who are not accustomed to being asked about their experiences or emotions
